# The usefulness of C-reactive protein and procalcitonin to predict prognosis in septic shock patients: A multicenter prospective registry-based observational study

**DOI:** 10.1038/s41598-019-42972-7

**Published:** 2019-04-29

**Authors:** Seung Mok Ryoo, Kap Su Han, Shin Ahn, Tae Gun Shin, Sung Yeon Hwang, Sung Phil Chung, Yoon Jung Hwang, Yoo Seok Park, You Hwan Jo, Hyung Lan Chang, Gil Joon Suh, Kyoung Min You, Gu Hyun Kang, Sung-Hyuk Choi, Tae Ho Lim, Won Young Kim, Korean Shock Society (KoSS) Investigators, Youn-Jung Kim, Sung Woo Lee, Ik Joon Jo, Min Joung Kim, Woon Yong Kwon, Hui Jai Lee, Jong Hwan Shin, Byuk Sung Ko

**Affiliations:** 1 Department of Emergency Medicine, University of Ulsan College of Medicine, Asan Medical Center, Seoul, Korea; 2 Department of Emergency Medicine, College of Medicine, Korea University, Anam Hospital, Seoul, Republic of Korea; 3 Department of Emergency Medicine, Samsung Medical Center, Sungkyunkwan University School of Medicine, Seoul, Republic of Korea; 4 Department of Emergency Medicine, Yonsei University College of Medicine, Seoul, Republic of Korea; 5 Department of Emergency Medicine, Seoul National University Bundang Hospital, Seongnam, Republic of Korea; 6 Department of Emergency Medicine, Seoul National University College of Medicine, Seoul, Republic of Korea; 7 Department of Emergency Medicine, Seoul National University Boramae Medical Center, Seoul, Korea; 8 Department of Emergency Medicine, Hallym University College of Medicine, Seoul, Republic of Korea; 9 Department of Emergency Medicine, College of Medicine, Korea University, Guro Hospital, Seoul, Republic of Korea; 10 Department of Emergency Medicine, College of Medicine, Hanyang University, Seoul, Republic of Korea

**Keywords:** Prognostic markers, Bacterial infection

## Abstract

The objective of this study was to evaluate the prognostic value of C-reactive protein (CRP), procalcitonin (PCT), and their combination for mortality in patients with septic shock. This multicenter, prospective, observational study was conducted between November 2015 and December 2017. A total of 1,772 septic shock patients were included, and the overall 28-day mortality was 20.7%. Although both CRP and PCT were elevated in the non-survivor group, only CRP had statistical significance (11.9 mg/dL vs. 14.7 mg/dL, p = 0.003, 6.4 ng/mL vs. 8.2 ng/mL, p = 0.508). Multivariate analysis showed that CRP and PCT were not independent prognostic markers. In the subgroup analysis of the CRP and PCT combination matrix using their optimal cut-off values (CRP 14.0 mg/dL, PCT 17.0 ng/dL), both CRP and PCT elevated showed significantly higher mortality (Odds ratio 1.552 [95% Confidence intervals 1.184–2.035]) than both CRP and PCT not elevated (p = 0.001) and only PCT elevated (p = 0.007). However, both CRP and PCT elevated was also not an independent predictor in multivariate analysis. Initial levels of CRP and PCT alone and their combinations in septic shock patients had a limitation to predict 28-day mortality. Future research is needed to determine new biomarkers for early prognostication in patients with septic shock.

## Introduction

Sepsis is a life threatening organ dysfunction evoked by abnormal host response to infection, and the Sequential Organ Failure Assessment (SOFA) score is used to calculate the degree of organ dysfunction in sepsis^1,2^. Septic shock is defined as a subset of sepsis in which underlying circulatory and cellular metabolism abnormalities are profound enough to substantially increase mortality^2^. We can evaluate the degree of shock by measuring mean arterial blood pressure as a circulatory abnormality and serum lactate level as a cellular metabolic abnormality^3^. However, proven biomarkers that reflect the severity of infection in patients with sepsis have not yet been identified, and current guidelines by the Surviving Sepsis Campaign do not provide any biomarkers that can evaluate or identify an infection, except procalcitonin (PCT) for indicating the timing of de-escalating antibiotics^3^.

C-reactive protein (CRP) is a traditional biomarker which is elevated in inflammatory states including rheumatoid arthritis and infection^4^. Aside from its roles as a biomarker, CRP also functions as a part of the defense mechanism against inflammation and pathogen invasion^5^. However, CRP has low specificity for diagnosing sepsis, and the plasma level of CRP is not a reliable indicator for the degree of systemic inflammation^6^.

PCT is used as an indicator for antibiotics treatment because the level of PCT is higher in fungal, parasitic, and bacterial infections than in viral infections. Accordingly, high early levels of PCT in sepsis have been suggested to be associated with unfavorable prognosis^7^. Nevertheless, early PCT levels are subject to alteration by the type and severity of the initial cause of the sepsis and not necessarily the severity of the sepsis itself; therefore, it is not recommended to utilize early PCT level as a definite indicator of prognosis^8^.

Research about the diagnostic ability of CRP and PCT has been conducted in sepsis, but data on the early prognostic value of CRP and PCT are lacking. This study evaluated the prognostic values of CRP and PCT for mortality prediction in septic shock cases.

## Results

**Figure 1:**
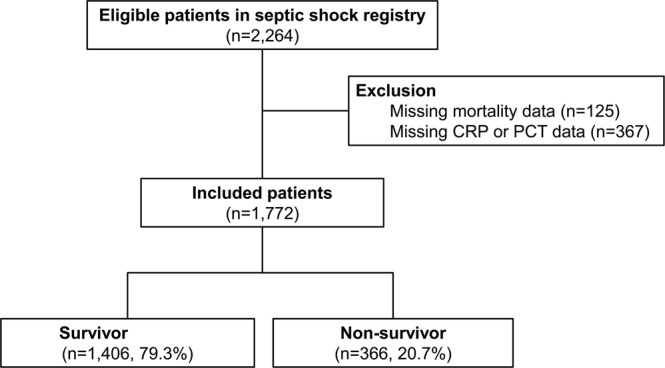
Diagram of included patients. Abbreviations: CRP, C-reactive protein; PCT, Procalcitonin.

Of the 2,264 eligible patients in the Korean Shock Society (KoSS) septic shock registry, we excluded 124 patients due to missing 28-day mortality data and 367 patients due to missing CRP or PCT data. Finally, 1,772 patients were included and divided into 1,406 (79.3%) survivors and 366 (20.7%) non-survivors. (Fig. 1)

The non-survivor group was male predominant (63.7% vs. 57.8% p = 0.041). Subjects were older (70.1 ± 13.0 years vs. 66.8 ± 13.7 years, p < 0.001) and had more co-morbidities, including diabetes and chronic pulmonary disease (35.5% vs. 28.5%, p = 0.009; 12.3% vs. 7.0%, p = 0.001; respectively), than the survivors. Their initial heart rate was faster (112.2 ± 25.0 vs. 104.2 ± 23.0, p < 0.001). Pneumonia was more frequent in the non-survivors (48.1% vs. 27.3%, p < 0.001); however, urinary tract infection and hepatobiliary and pancreas infection were more common in the survivors (19.1% vs. 26.7%, p = 0.001; 16.4% vs. 21.1%, p = 0.048; respectively). The severity scores, including maximum SOFA and Acute Physiology and Chronic Health evaluation (APACHE) II scores were higher in the non-survivor group (10.0 [8.0–13.0] vs. 7.0 [5.0–10.0], p < 0.001; 24.0 [18.0–34.0] vs. 18.0 [13.0–24.0], p < 0.001; respectively) (Table 1).

**Table 1:** Baseline and clinical characteristics of the study population grouped into survivors and non-survivors.

Characteristics	Total (n = 1,772)	Survivors (n = 1,406)	Non-survivors (n = 366)	p-value
Age, years	67.5 ± 13.6	66.8 ± 13.7	70.1 ± 13.0	<0.001
Male	1,045 (59.0)	812 (57.8)	233 (63.7)	0.041
**Past medical history**				
Hypertension	723 (40.8)	560 (39.8)	163 (44.5)	0.103
Diabetes	531 (30.0)	401 (28.5)	130 (35.5)	0.009
Coronary artery disease	226 (12.8)	177 (12.6)	49 (13.4)	0.683
Stroke	211 (11.9)	160 (11.4)	51 (13.9)	0.179
Chronic pulmonary disease	144 (8.1)	99 (7.0)	45 (12.3)	0.001
Metastatic cancer	434 (24.5)	331 (23.5)	103 (28.1)	0.068
**Vital signs at shock recognition**				
Systolic blood pressure, mmHg	89.4 ± 23.1	89.1 ± 22.3	90.4 ± 26.1	0.384
Diastolic blood pressure, mmHg	54.3 ± 16.0	53.7 ± 14.9	56.4 ± 19.6	0.016
Pulse rate, beats/min	105.8 ± 23.6	104.2 ± 23.0	112.2 ± 25.0	<0.001
**Infection focus**				
Pneumonia	560 (31.6)	384 (27.3)	176 (48.1)	<0.001
Urinary tract infection	446 (25.2)	376 (26.7)	70 (19.1)	0.001
Hepatobiliary and pancreas infection	356 (20.1)	296 (21.1)	60 (16.4)	0.048
Gastrointestinal infection	303 (17.1)	230 (16.4)	73 (19.9)	0.104
**Laboratory findings**				
White blood cell count (×10^3^/μL)	10.2 [5.1–16.6]	10.2 [5.3–16.5]	9.8 [4.2–17.0]	0.342
Hemoglobin, g/dL	11.0 ± 2.5	11.1 ± 2.4	10.7 ± 2.8	0.003
Creatinine, mg/dL	1.3 [0.9–2.2]	1.3 [0.9–2.0]	1.6 [1.0–2.6]	<0.001
Blood urea nitrogen, mg/dL	26.6 [18.0–40.4]	25.5 [17.0–37.0]	33.0 [21.7–49.0]	<0.001
Aspartate transaminase, IU/L	39.0 [24.0–82.0]	38.0 [24.0–76.0]	44.5 [27.0–105.0]	0.001
Alanine transaminase, IU/L	25.0 [14.0–54.0]	26.0 [15.0–52.0]	25.0 [14.0–58.3]	0.825
Initial lactate level, mmol/L	3.3 [1.9–5.4]	3.0 [1.8–4.9]	4.9 [2.7–8.3]	<0.001
C-reactive protein, mg/dL	12.3 [4.6–21.8]	11.9 [4.3–21.0]	14.7 [5.8–25.1]	0.003
Procalcitonin, ng/mL	6.8 [1.1–27.6]	6.4 [1.0–26.8]	8.2 [1.1–30.7]	0.508
**Severity score**				
Maximum SOFA	8.0 [5.0–11.0]	7.0 [5.0–10.0]	10.0 [8.0–13.0]	<0.001
APACHE-II score	19.0 [14.0–26.0]	18.0 [13.0–24.0]	24.0 [18.0–34.0]	<0.001

Values were expressed as means ± standard deviation, medians [interquartile range], or numbers (%) Abbreviations: SOFA, Sequential Organ Failure Assessment; APACHE, Acute Physiology and Chronic Health Evaluation.

Serum creatinine, blood urea nitrogen, aspartate transaminase, and initial lactate levels were significantly higher in the non-survivors (1.3 [0.9–2.0] vs. 1.6 [1.0–2.6], p < 0.001; 25.5 [17.0–37.0] vs. 33.0 [21.7–49.0], p < 0.001; 38.0 [24.0–76.0] vs. 44.5 [27.0–105.0], p = 0.001; 3.0 [1.8–4.9] vs. 4.9 [2.7–8.3], p < 0.001; respectively). However white blood cell count was not different (10.2 [5.3–16.5] vs. 9.8 [4.2–17.0], p = 0.342; Table 1). The initial PCT level also did not differ significantly (odds ratios (OR) 0.999 [95% confidence intervals (CI) 0.997–1.001]); however, initial CRP level was significantly higher in the non-survivors (OR 1.013 [95% CI 1.004–1.022]). Although in univariate logistic regression, CRP increased the 28-day mortality rate it was not an independent predictor of 28-day mortality in multivariate logistic regression analysis (Table 2). The optimal cut-off values of CRP and PCT in receiver operating characteristic (ROC) curve were 14 mg/dL and 17 ng/dL, respectively. The sensitivity, specificity, positive predictive value, and negative predictive value of CRP were 52.5%, 56.4%, 23.9%, and 82.0%, respectively, and those of PCT were 39.1%, 65.7%, 22.8%, and 80.5, respectively.

**Table 2:** Univariate and multivariate analysis for 28-day mortality.

Characteristics	Univariate OR [95% CI]	Multivariate OR [95% CI]	p-value
Age, years	1.019 [1.010–1.028]	1.014 [1.003–1.025]	0.009
Chronic lung disease	1.851 [1.275–2.687]		
Pulse rate, beats/min	1.014 1.009–1.019]		
Pneumonia	2.465 [1.947–3.122]	2.113 [1.609–2.775]	<0.001
Urinary tract infection	0.648 [0.487–0.862]		
Maximum SOFA	1.242 [1.201–1.284]	1.163 [1.109–1.220]	<0.001
APACHE-II	1.089 [1.074–1.103]	1.025 [1.006–1.044]	0.008
Creatinine, mg/dL	1.150 [1.072–1.234]	0.876 [0.777–0.988]	0.031
Blood urea nitrogen, mg/dL	1.016 [1.011–1.021]		
Aspartate transaminase, IU/L	1.000 [1.000–1.001]		
Initial lactate level, mmol/L	1.211 [1.169–1.254]	1.163 [1.119–1.208]	<0.001
C-reactive protein, mg/dL	1.013 [1.004–1.022]		
Procalcitonin, ng/mL	0.999 [0.997–1.001]		

Logistic regression analysis with backward elimination method.

Abbreviations: OR, odds ratio; CI, confidence interval; SOFA, Sequential Organ Failure Assessment; APACHE, Acute Physiology and Chronic Health Evaluation.

**Figure 2:**
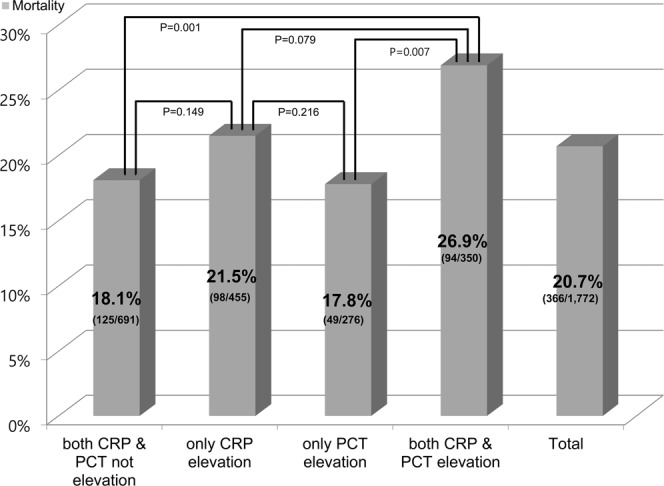
Mortality of procalcitonin (PCT), C-reactive protein (CRP), and their combinations. Optimal cut off of CRP elevation was defined as ≥14 mg/dL and PCT elevation was defined as ≥17 ng/mL. Overall 28-day mortality was 20.7%. The mortality rates of both CRP and PCT not elevated, only CRP elevated, only PCT elevated, and both CRP and PCT elevated were 18.1%, 21.5%, 17.8%, and 26.9%, respectively. The mortality of both CRP and PCT elevated was significantly higher than for both not elevated and only PCT elevated.

The combination matrix of CRP and PCT was compared to determine the 28-day mortality of each group (Fig. 2). The OR of both CRP and PCT elevated was 1.552 (95% CI 1.184–2.035), and the mortality rate was 26.9%. The 28-day mortality of both CRP and PCT elevated was significantly higher than that of only PCT elevated (17.8%) and both CRP and PCT not elevated (18.1%). However, the 28-day mortality of patients with only CRP elevated was 21.5% which was not significantly different from those with both CRP and PCT elevated (p = 0.079) (Fig. 2, Table 3). Nevertheless, in the multivariate logistic regression analysis, both CRP and PCT elevated was not an independent predictor of 28-day mortality.

**Table 3:** Baseline and clinical characteristics of the study population using optimal cut-off values for C-reactive protein and procalcitonin.

Characteristics	Both CRP and PCT not elevated (n = 691)	Only CRP elevated^a^ (n = 455)	Only PCT elevated^b^ (n = 276)	Both CRP and PCT elevated (n = 350)	p-value
Age, years	66.9 ± 13.9	68.9 ± 13.0	67.8 ± 13.9	66.7 ± 13.7	0.054
Male	415 (60.1)	265 (58.2)	180 (65.2)	185 (52.9)	0.016
**Past medical history**					
Hypertension	247 (35.7)	194 (42.6)	116 (42.0)	166 (47.4)	0.002
Diabetes	202 (29.2)	119 (26.2)	89 (32.2)	121 (34.6)	0.056
Coronary artery disease	105 (15.2)	53 (11.6)	33 (12.0)	35 (10.0)	0.081
Stroke	87 (12.6)	56 (12.3)	26 (9.4)	42 (12.0)	0.571
Chronic pulmonary disease	57 (8.2)	45 (9.9)	16 (5.8)	26 (7.4)	0.246
Metastatic cancer	162 (23.4)	119 (26.2)	68 (24.6)	85 (24.3)	0.777
**Vital signs at shock recognition**					
Systolic blood pressure, mmHg	91.8 ± 24.0	92.3 ± 24.1	83.0 ± 17.5	85.7 ± 22.6	<0.001
Diastolic blood pressure, mmHg	54.7 ± 15.9	55.5 ± 16.9	51.7 ± 12.0	53.9 ± 17.6	0.016
Pulse rate, beats/min	103.6 ± 23.7	106.9 ± 23.0	105.0 ± 22.3	109.5 ± 24.1	0.001
**Infection focus**					
Pneumonia	223 (32.3)	183 (40.2)	54 (19.6)	100 (28.6)	<0.001
Urinary tract infection	142 (20.5)	103 (22.6)	85 (30.8)	116 (33.1)	<0.001
Hepatobiliary and pancreas infection	133 (19.2)	74 (16.3)	76 (27.5)	73 (20.9)	0.003
Gastrointestinal infection	136 (19.7)	69 (15.2)	46 (16.7)	52 (14.9)	0.126
**Laboratory findings**					
White blood cell count (×10^3^/μL)	9.6 [5.1–15.3]	11.6 [5.7–17.9]	9.5 [4.5–17.0]	10.5 [4.4–17.8]	0.058
Hemoglobin, g/dL	11.1 ± 2.5	10.7 ± 2.4	11.4 ± 2.7	11.2 ± 2.6	0.001
Creatinine, mg/dL	1.1 [0.8–1.6]	1.2 [0.9–2.0]	1.7 [1.2–2.5]	2.1 [1.4–2.9]	<0.001
Blood urea nitrogen, mg/dL	21.2 [15.0–31.0]	26.0 [18.7–39.0]	29.0 [20.8–40.3]	38.0 [28.0–51.9]	<0.001
Aspartate transaminase, IU/L	35.0 [23.0–69.3]	35.0 [23.0–60.0]	58.0 [28.0–148.0]	45.0 [29.0–93.3]	<0.001
Alanine transaminase, IU/L	23.0 [14.0–49.0]	23.0 [13.0–43.0]	35.5 [16.0–89.8]	29.0 [16.0–59.0]	<0.001
Initial lactate level, mmol/L	2.9 [1.7–5.0]	2.8 [1.7–4.7]	4.0 [2.4–6.5]	4.3 [2.7–6.4]	<0.001
CRP, mg/dL	5.1 [1.2–9.4]	22.0 [17.5–29.3]	5.5 [0.7–9.9]	25.0 [18.8–30.3]	<0.001
PCT, ng/mL	1.2 [0.4–5.4]	3.3 [1.0–8.1]	41.8 [24.5–66.7]	46.0 [25.6–94.9]	<0.001
**Severity score**					
Maximum SOFA	7.0 [5.0–10.0]	7.0 [5.0–10.0]	9.0 [6.0–12.0]	10.0 [7.0–12.0]	<0.001
APACHE-II score	18.0 [12.0–24.0]	19.0 [14.0–26.0]	19.0 [13.3–26.0]	22.0 [15.0–28.0]	<0.001
28-day mortality	125 (18.1)	98 (21.5)	49 (17.8)	94 (26.9)	0.006

Values were expressed as means ± standard deviation, medians [interquartile range], or numbers (%).

^a^CRP elevation ≥14.0 mg/dL.

^b^PCT elevation ≥17.0 ng/mL Abbreviations: CRP, C-reactive protein; PCT, procalcitonin; SOFA, Sequential Organ Failure Assessment; APACHE, Acute Physiology and Chronic Health Evaluation.

## Discussion

In our study, the initial levels of CRP and PCT did not show significant association with 28-day mortality. When we compared the combination matrix of CRP and PCT using the optimal cut-off values, the mortality rate of patients with elevated CRP and PCT was significantly higher than that of patients with non-elevated CRP and PCT or only elevated PCT. However, both CRP and PCT elevated was not an independent predictor of 28-day mortality.

Sepsis is an infectious condition associated with organ dysfunction; therefore, a diagnosis of infection is crucial. Most sepsis-related infection symptoms and variables such as fever and tachycardia are not necessarily sepsis-specific. Therefore, in order to increase the accuracy of sepsis diagnosis, physicians should survey for other indicators such as biomarkers (e.g., CRP, PCT), imaging data (e.g., X-ray), and organ dysfunction^9^. A previous study showed that patients with mixed bacterial pneumonia had significantly higher serum levels of CRP and PCT than in those with viral infection alone^10^. Another study in neonatal sepsis patients also reported that both CRP and PCT showed relatively good performance in discriminating proven sepsis from controls, and their combination increased the accuracy of neonatal sepsis diagnoses^11^. However, the primary outcome of both previous studies was diagnosis of infection and sepsis. The early prognostic value of CRP and PCT in septic shock cases that received protocol-driven resuscitation bundle therapy at emergency departments (EDs) remains unclear. Our current data show that CRP, PCT, and their combination have limited ability in predicting mortality in septic shock cases. CRP was associated with 28-day mortality in univariate models only and not in the multivariate model. We speculate that initial CRP and PCT might be measured too early to reflect disease severity. However, that initial levels of CRP and PCT were obtained before resuscitation, specifically before antibiotics administration which can influence CRP and PCT levels, could be considered strength of this study. PCT induction occurs at approximately 2–4 hours after the onset of sepsis, and peaks at 24–48 hours^6^. Thus, because CRP elevation is also expected to occur 24–48 hours after the initial inflammatory response^4^, initial CRP and PCT may not be considered to be useful markers in patients with acute and critical conditions.

A systemic review and meta-analysis study showed that PCT levels were significantly different between surviving and non-surviving sepsis patients^12^. Among 25 analyzed studies, only four were conducted at EDs. Because most studies were conducted at intensive care units (ICUs), the measured time of PCT is doubtful. Additionally, the heterogeneity among the studies was very high. The mortality reported in the studies ranged from 13% to 69%, and the severity of infection ranged from sepsis to septic shock. Another recent study also reported that the levels of PCT and CRP at admission showed association with mortality. However, their obtained sample time was the morning after admission, and mortality was excessively high, sepsis was 43.33%, and septic shock was 75%^13^. In contrast, a recent study performed in pediatrics reported that median CRP and PCT were not associated with mortality; overall mortality was 33.3%, and samples were obtained on the first day of ICU admission^14^.

Because of these varying results, other studies using biomarkers have been conducted to predict prognosis. Huang *et al*. studied PCT clearance. In their study, although PCT levels on days 1, 3, and 5 were not associated with prognosis, PCT clearance rates at days 3 and 5 were significantly higher in the survivor group^15^. Another study also evaluated the PCT trend and reported that a decrease in the initial PCT level of more than 80% on day 4, rather than initial PCT level, was predictive for survival^16^. Hahn *et al*. demonstrated a PCT to CRP ratio to detect late onset neonatal sepsis. In their study, the area under the curve of PCT/CRP was 0.73 for distinguishing between proven and suspected sepsis^11^. However, this study did not report the prognostic value of the PCT/CRP ratio.

In our study, we evaluated the combination of CRP and PCT for their prognostic power in predicting mortality. We found that patients who had elevation of both CRP and PCT had significantly higher mortality rate than did the other groups, except those who had elevation of CRP only. Because CRP is associated with immunity^4^ and reflects an inflammatory condition^5^ which is associated with the host defense mechanism, it might more directly influence mortality, whereas PCT is a very sensitive biomarker of bacterial infection^6^, Moreover, antibiotics administration time could confound the spread of bacterial infection therefore, it might confound the initial PCT value. If so, the PCT variation trend, rather than initial value, may be more associated with prognosis as shown previously^16^. In this study, because of the nature of a prospective multi-center observational study, we were unable to determine a trend for PCT. In contrast, previous studies reported that several days were needed for the CRP levels to peak, whereas PCT levels peaked at 24–48 hours after sepsis, despite induction after approximately 2–4 hours^6^. Therefore, initial CRP and PCT levels in this study might be underestimated.

As is well known, PCT and CRP are suitable markers for the diagnosis of sepsis, and they have been used for the early detection of infection and guiding of antibiotics therapy. However, in this study we were unable to confirm a limited ability to predict mortality in septic shock patients, specifically considering initial CRP and PCT levels. Although infection is a cause of sepsis, the degree of shock or organ failure as a result of septic shock may be more suitable for predicting mortality. Future research will most likely focus on the development of new biomarkers or combinations of markers with clinical signs for predicting prognosis of early septic shock and guiding therapy.

This study had some limitations. Due to its prospective observational design, we were unable to obtain additional laboratory data such as repeated CRP and PCT levels. Analyzing the biomarkers several days after sepsis development may show an association with mortality. However, in the clinical setting, because factors are required which can predict mortality early-on, biomarkers obtained several days after diagnosis are not helpful. Moreover, during septic shock management various factors influence mortality; therefore, more valuable results may be obtained if possible risk factors can be controlled.

CRP was more predictive than PCT, and elevation of both CRP and PCT was associated with the highest mortality rate among all combinations. However, CRP and PCT alone as well as their combination were not independent predictors of 28-day mortality in septic shock cases. Further studies are needed to identify the biomarkers for early prognostication in patients with septic shock.

## Methods

### Setting and study population

This was a multicenter prospective, observational, registry-based study using KoSS septic shock registry data. The KoSS, a multicenter clinical research consortium for septic shock, was organized in 2013, and KoSS investigators have been prospectively collecting data from septic shock patients at the EDs of 10 teaching hospitals throughout South Korea from October 2015. The institutional review board of Asan Medical Center [2015–1253] and each institution (Korea University Anam Hospital [HRPC2016-184], Samsung Medical Center [SMC2015-09-057], Yonsei University College of Medicine Severance Hospital [4-2015-0929], Gangnam Severance Hospital [3-2015-0227], Seoul National University Bundang Hospital [B-1409/266-401], Seoul National University College of Medicine [J-1408-003-599], Seoul National University Boramae Medical Center [16-2014-36], Hallym University College of Medicine Gangnam Secred Heart Hospital [2015-11-142], Korea University Guro Hospital [KUGH15358-001], Hanyang University Hospital [HYUH2015-11-013-007]) approved the study protocol, and informed consent was obtained before data collection. All experiments were performed in accordance with relevant guidelines and regulations^17^.

Adult (≥18 years) septic shock patients, defined by suspected or confirmed infection and evidence of refractory hypotension or hypoperfusion, were enrolled in the registry^18–20^. Refractory hypotension was defined as persistent hypotension which was systolic blood pressure (SBP) <90 mm Hg, a mean arterial pressure <70 mm Hg, or an SBP decrease of >40 mm Hg after adequate intravenous fluid challenge (20–30 mL/kg or at least 1 L or more of crystalloid solution administered over 30 minutes), or as the need for vasopressors after fluid resuscitation^21^. Hypoperfusion was defined as a serum lactate concentration of ≥4 mmol/L^22^. Patients who signed a “Do Not Attempt Resuscitation” order, met the inclusion criteria six hours after ED arrival, were transferred from other hospitals without meeting the inclusion criteria upon ED arrival, or were directly transferred from an ED to other hospitals were not enrolled in in the KoSS septic shock registry. The case report form, standard definitions of 200 variables including clinical characteristics, therapeutic interventions, and outcomes of patients with septic shock, and an investigator manual were developed based on a literature review and consensus by the study investigators. Data were collected via a standardized registry form and entered into a web-based electronic database registry. Outliers or incorrect values are primarily filtered by this data entry system. Additionally, the principal investigator of each site has a designated local research coordinator who is responsible for ensuring the accuracy of data and verifying records. The quality management committee (QMC), which consists of emergency physicians, local research coordinators, and investigators in each ED, monitors and reviews data quality regularly. The QMC gives feedback to each research coordinator and investigator of the results of the quality management process through the query function in the system or directly by phone to clarify data^17^.

### Data collection

All KoSS septic shock registry data were collected from November 2015 to December 2017. Demographic and clinical data, including age, gender, previous medical history, initial vital signs, severity, and laboratory values on admission, and interventions were retrieved from the septic shock registry. Maximum SOFA and APACHE-II scores were evaluated using the worst parameters within 24 hours after ED arrival^17,23^.

All laboratory parameters, including CRP and PCT levels, were measured at the ED upon initial septic shock recognition. We determined the cut-off values of CRP and PCT to predict 28-day mortality using the Youden Index and divided patients into subgroups as follows: both CRP and PCT not elevated, only CRP or PCT elevated, and both CRP and PCT elevated.

The primary clinical outcome of this study was the 28-day mortality rate. We evaluated the predictive ability of CRP, PCT, and their combination for 28-day mortality rates.

### Statistical analyses

Continuous variables were expressed as means ± standard deviation or medians with the interquartile range if the assumption of a normal distribution was violated. Categorical variables were expressed as numbers and percentages. To analyze the baseline characteristics and laboratory examinations in survivor and non-survivor groups, Student’s *t*-test was used to compare the means of normally distributed continuous variables; the Mann-Whitney U test was used to compare non-continuous variables. The Chi-squared or Fisher’s exact test was used to compare categorical variables^17^. The optimal cut-off value of CRP was predicted by the Youden Index using ROC curve analysis in a univariate model. Multivariate analyses were performed using a logistic regression with a backward elimination method to evaluate the association between the clinical factors, including laboratory data and 28-day mortality. The results of the multivariate logistic regression were reported as OR and 95% CI. We conducted an Analysis of Variance and Kruskal-Wallis analysis to evaluate differences between subgroup analyses.

All tests in this study were two-sided, and a p-value < 0.01 was considered to be statistically significant. All statistical analyses were performed using SPSS for Windows version 20.0 (SPSS Inc., Chicago, IL, USA).
